# Deep Genomic Sequencing of Bladder Urothelial Carcinoma in Southern Chinese Patients: A Single-Center Study

**DOI:** 10.3389/fonc.2021.538927

**Published:** 2021-05-14

**Authors:** Dong-Yang Li, Fei Yang, Wei-Qiang Liao, Xiang-Fu Zhou, Wen-Biao Li, Jia-Rong Cai, Bo-Long Liu, Yun Luo, Hai-Lun Zhan

**Affiliations:** ^1^ Department of Urology, The Third Affiliated Hospital of Sun Yat-Sen University, Guangzhou, China; ^2^ Department of Urology, Luoding People’s Hospital, Luoding, China

**Keywords:** deep sequencing, genomic alteration, Chinese, bladder urothelial carcinoma, tumor mutation burden

## Abstract

**Objective:**

Bladder urothelial carcinoma (BUC) is a common urological malignancy with molecular heterogeneity. However, the genetic feature of Chinese BUC patients is still not well-identified.

**Methods:**

We performed deep sequencing by a large panel (450 genes) on 22 BUC samples and using matched normal bladder tissue as control. Genomic alterations (GAs), pathways and Tumor Mutation Burden (TMB) were investigated.

**Results:**

The frequencies of GAs (*TERT*, 54.5%; *CREBBP*, 27.3%; *GATA3*, 22.7%; *BRAF*, 18.2%; *TEK*, 18.2% and *GLI1*, 18.2%) were significantly higher in Chinese than Western BUC patients. Other GAs’ frequencies were in accordance with previous study (*TP53*, 50.0%; *KDM6A*, 31.8%; *KMT2D*, 22.7%; etc.). Besides, we detected gene amplification in *ERBB2, FRS2, FAS*, etc. The gene fusion/rearrangement took place in the chromosome 11, 12, 14, 17, 19, 22, and Y. Other than cell cycle and PI3K-AKT-mTOR, mutated genes were more associated with the transcription factor, chromatin modification signaling pathways. Interestingly, the TMB value was significantly higher in the BUC patients at stages T1–T2 than T3–T4 (*P* = 0.025).

**Conclusion:**

Deep genomic sequencing of BUC can provide new clues on the unique GAs of Chinese patients and assist in therapeutic decision.

## Introduction

Bladder cancer (BCa) is a common malignancy among urological cancers, ranking only second to prostate cancer (1). The American Cancer Society estimated that there would be 80,470 new BCa cases and 12,870 deaths in the United States in 2019 (2). Approximately 90% histological subtype of BCa is bladder urothelial carcinoma (BUC) (3). Non-muscle invasive bladder cancer (NMIBC) accounts for nearly 75% of BCa at diagnosis and transurethral resection of bladder tumor (TURBT) is usually the preferred treatment (4). Even though traditional treatment strategies such as surgery, instillation chemotherapy or vaccine therapy can be applied, up to 70% NMIBC will recur within 2 years (5). In addition, about 1–45% NMIBC cases will infiltrate deeper and progress to muscle invasive bladder cancer (MIBC) in 5 years (6). Thus, novel and individualized therapies are demanded for the patients with BCa.

In the past decades, immunotherapy of cancer has been developing rapidly. The immunotherapy categories generally contain monoclonal antibody, tumor vaccine, engineered chimeric antigen receptor (CAR) T cells and immune cells modulator. Recently, the immune checkpoint inhibitors (ICIs), such as cytotoxic T-lymphocyte antigen 4 (CTLA-4) blocking antibodies and anti-programmed death-1 (PD-1), have experienced remarkable advances in the clinical application of melanoma, non-small cell lung cancer, etc. (7). Clinical trials showed the striking effects of ICIs in locally advanced and metastatic BCa, however, because of genomic heterogeneity, only 20% BCa patients can benefit from ICIs (8, 9). Hence, precision medicine calls for the transition to targeted therapy. Next generation sequencing can facilitate urologists to identify specific clinically relevant gene mutation and formulate corresponding treatment measures.

In the present study, genomic alterations (GAs) were investigated in 22 BUC samples and paired normal bladder tissues/peripheral blood samples by a 450-gene sequencing panel. The different GAs were compared between southern Chinese BUC patients and Western BUC patients. The tumor mutation burden (TMB) was also calculated, and the correlations of TMB and GAs with clinical factors were analyzed. To the best of our knowledge, there is still no literature about the large panel genomic sequencing data on southern Chinese BUC patients. The purpose of this study was to further elucidate the GAs profile of southern Chinese BUC patients based on a single center.

## Patients And Methods

### Patients and Samples

A total of 22 southern Chinese patients who received TURBT or radical cystectomy in the third affiliated hospital of Sun Yat-Sen University from July, 2014 to January, 2019 were enrolled in this study. All the 22 patients were Han ethnic. All patients were diagnosed as BUC after surgery by two senior pathologists. None of these 22 patients had chemotherapy prior to sequencing. The TNM stages were determined according to the 8th American Joint Committee on Cancer (AJCC) staging system. Frozen or formalin-fixed paraffin-embedded (FFPE) cancer tissues and matched normal bladder tissues/matched peripheral blood samples from these 22 BUC patients were collected and sent to the laboratory of OrigiMed, (Shanghai, China) for GAs detection. If multiple bladder cancers occurred in one patient, only the highest histological grade cancer tissue was collected. This study was conducted in accordance with the Declaration of Helsinki. This study was approved by the Ethical Committee of the Third Affiliated Hospital of Sun Yat-Sen University (No. 2019-02-253-01) and written informed consent was obtained from each patient enrolled.

### Sequencing and Genomic Alterations Detection

At least 50 ng cancer tissue DNA was extracted from each cancer tissue by DNA Extraction Kit (Qiagen, Hilden, Germany) according to the manufacturer’s protocols. The GAs were measured by using the Yuan-Su450™ panel (OrigiMed, Shanghai, China), which covers all exons of 450 cancer-related genes and 39 selected genes’ introns that frequently take part in rearrangement. This panel contains genes which were related to targeted drugs, immunotherapy, chemoradiotherapy, genetic risks, and other cancer-related genes of cancer signal pathways, epigenetic modulation and prognosis. In addition, this panel is benefit for patients in the clinical practice and costs less than the whole genomic sequencing. For the low read depths region, the probe density of capture panel was increased to guarantee reliability. The DNA libraries were then sequenced with 900× mean coverage for cancer and matched normal tissues/matched blood samples on an Illumina NextSeq-500 platform (Illumina, San Diego, USA). The data processing pipeline in this study contained pre-prossing, alignment, detection and annotation (10). The strictly quality control for each step was launched (11–13). The GAs include single nucleotide variation (SNV, truncation, substitution), copy number variation (CNV), long/short fragment indel/deletion and gene rearrangement/fusion. Although the sequencing results were not validated by Sanger or qPCR, all of the detected mutations were compared with the in-house database (OrigiMed) of genomic changes specific to clinical annotation. A suite of bioinformatics pipelines and manual review were performed to ensure no false positives or mistakes (14).

### Statistical Analysis

Statistical analyses were performed by using the SPSS 22.0 software (IBM, Chicago, IL, USA). The genetic mutation data of 410 Western BUC patients (The Cancer Genome Altas, TCGA) were acquired from the cbioportal website (www.cbioportal.org) (15). Differences between groups were tested by Fisher’s exact test (n < 40). For the comparison of quantitative variables, the Student’s *t*-test was used. It was defined as statistically significance when the *P* value < 0.05.

## Results

### Characteristics of Participants

Twenty male and two female BUC patients were enrolled in this study. The median age of these patients was 68.5 years (ranging from 56 to 86 years). All the participants possessed the genomic feature of microsatellite stable. Staging, grade, tumor mutation burden (TMB) and other clinical data are listed in **Table 1**.

**Table 1:** Characteristics of participants with bladder urothelial carcinoma.

Characteristics	Number	Percentage
Age		
<65 years	8	36.4
>65 years	14	63.6
Sex		
Male	20	90.9
Female	2	9.1
Subtype		
NMIBC	14	63.6
MIBC	8	36.4
Histological Grade		
Low grade	7	31.8
High grade	15	68.2
MSI status		
MSS	22	100.0
T stage		
T1	14	63.6
T2	5	22.7
T3	2	9.1
T4	1	4.5
N stage		
N0	19	86.4
N1	2	9.1
N3	1	4.5
M stage		
M0	22	100.0
TMB status		
<10 muts/Mb	11	50.0
10–20 muts/Mb	6	27.3
>20 muts/Mb	5	22.7
Total	22	

NMIBC, non-muscle invasive bladder cancer; MIBC, muscle invasive bladder cancer; MSI, microsatellite instability; MSS, microsatellite stable; TMB, tumor mutation burden; muts, mutant numbers; Mb, mega byte; T, tumor; N, node; M, metastasis.

### GAs Profile of Chinese BUC Patients

**Figure 1:**
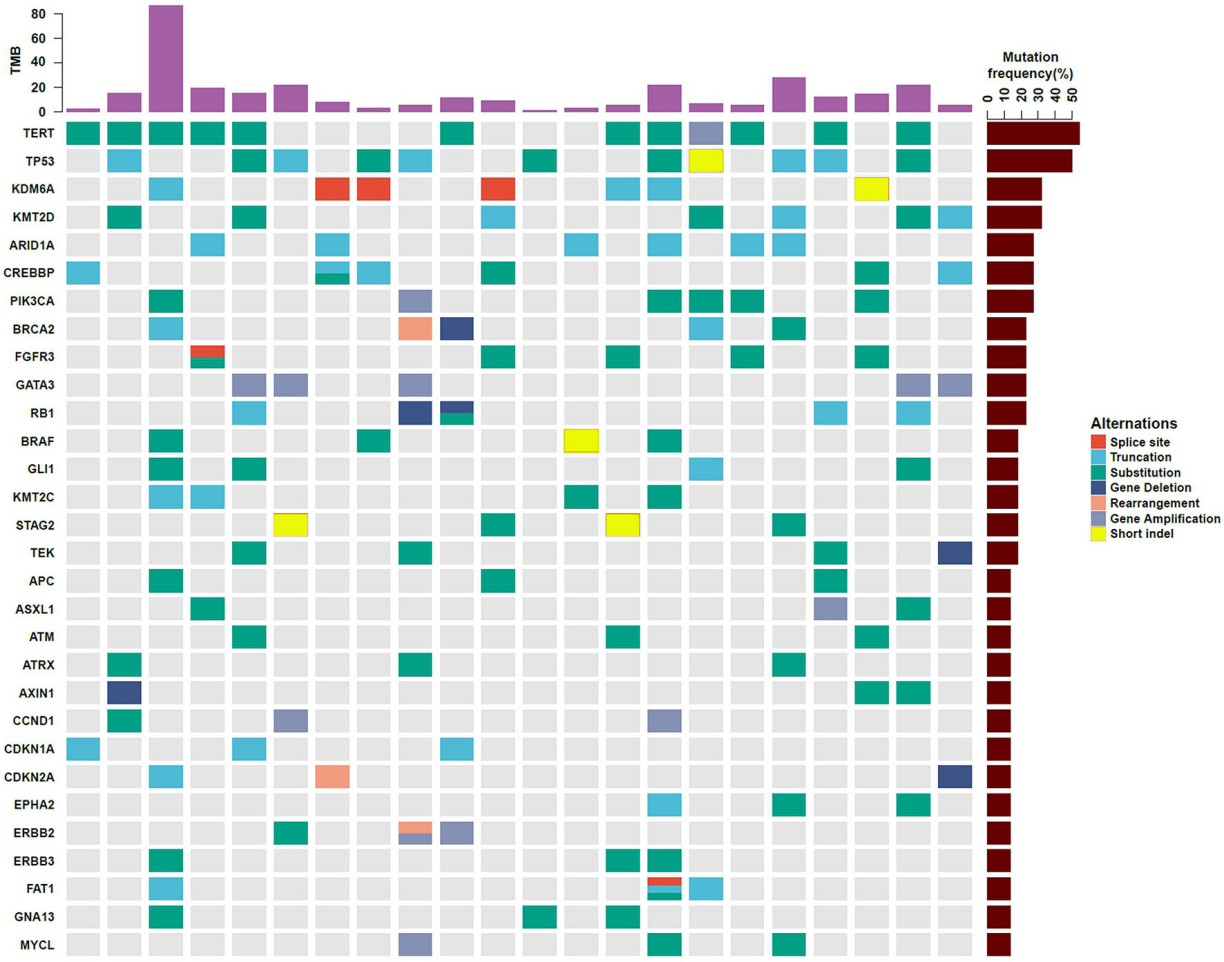
Gene mutation profiles of 22 southern Chinese patients with bladder urothelial carcinoma. The types of gene mutations are shown in different colors. TMB, tumor mutation burden.

All the 22 BUC patients were tested to possess clinical relevant GAs. As for a single patient, the number of mutated genes ranged from 2 to 45. The mutated genes profiles of the 22 participants are displayed in **Figure 1**. The most frequently found genes were *TERT* (n = 12, 54.5%), *TP53* (n = 11, 50.0%), *KDM6A* (n = 7, 31.8%), *KMT2D* (n = 7, 31.8%), *ARID1A* (n = 6, 27.3%), *CREBBP* (n = 6, 27.3%), *PI3KCA* (n = 6, 27.3%), *BRCA2* (n = 5, 22.7%), *FGFR3* (n = 5, 22.7%), *GATA3* (n = 5, 22.7%) and *RB1* (n = 5, 22.7%). Moreover, the alteration types were clearly shown in different colors, including splice site, truncation, substitution, deletion, rearrangement, amplification and short indel (**Figure 1**). The overall sequencing data of the 22 BUC patients were listed in **Supplementary Table 1**. The raw sequencing data of this study have been deposited into CNGB Sequence Archive (CNSA) of China National GeneBank DataBase (CNGBdb) with accession number CNP0001762 (16).

**Figure 2:**
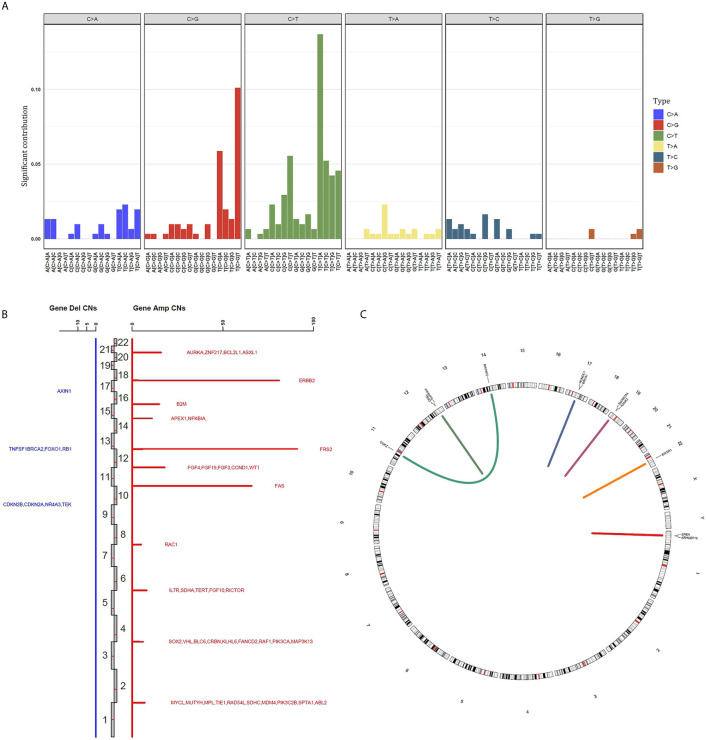
Summary of different genomic alteration types in 22 southern Chinese patients with bladder urothelial carcinoma. **(A)** The single nucleotide variation status in bladder urothelial carcinoma. **(B)** The genes with copy number variation are displayed in all chromosomes. The copy number of each mutant gene is the average number of mutated samples. Blue, gene deletion; Red, amplification; Del, deletion; Amp, amplification; CNs, copy numbers. **(C)** Gene fusion/rearrangement results of four patients. Patient 1: BRCA1-RUNDC1; patient 2: TBX3 intergenic; patient 3: EWSR1-SLC5A4; patient 4: MAP4K5-DGKZ, SMARCA4-KANK2 and SPEN-ARHGEF19.

The “C > T” dominated the different forms of SNV, accounting for 22.1% among the whole SNVs. Besides, the base “TCA > TTA” was the most frequent mode in “C > T” SNV (**Figure 2A** and **Supplementary Table 2**). Whereas the “T > G” ranked the last within the different forms of SNV. With regard to the CNV, the gene *FRS2* had the highest copy numbers (CNs, 91). The other two top amplified genes were *ERBB2* (CNs = 81), *FAS* (CNs = 66). While the following deleted genes were identified: *AXIN1*, *TNFSF1*, *BRCA2*, *FOXO1*, *RB1*, *CDKN2B*, *CDKN2A*, *NR4A3*, *TEK* (**Figure 2B** and **Supplementary Table 2**). Gene fusion/rearrangement was detected in four patients among the total 22 BUC participants. In addition, multiple gene rearrangements were found in only one patient. The gene fusion/rearrangement took place in chromosomes 11, 12, 14, 17, 19, 22, and Y (**Figure 2C**). The patient with multiple gene fusion/rearrangement was detected with *MAP4K5/DGKZ* in the chromosome 14, *SMARCA4/KANK2* in the chromosome 19 and *SPEN/ARHGEF19* in the chromosome Y (**Figure 2C**).

### Gene Mutations in Signaling Pathways

There were 82 mutated genes which occurred in at least two Chinese BUC patients. We subsequently summarized the relevant signal pathways based on these 82 genes. The five commonest signaling pathways in BUC were associated with transcription factor, chromatin modification, cell cycle, PI3K-AKT-mTOR and DNA repair. The mutated genes and number of cases were demonstrated in **Table 2**.

**Table 2:** The top five pathways and genes involved in Chinese patients with bladder urothelial carcinoma.

Pathway	Genes involved
Transcription factor	*TERT* (12, 22)*, CREBBP* (6, 22)*, GATA3* (5, 22)*, ASXL1* (3,22)*, ETV1* (2, 22)*, GLI3* (2, 22)*, NCOR1* (2, 22), *WT1* (2, 22)*, ZNF217* (2,22)*, FRS2* (2, 22)*, TBX3* (2, 22)*, U2AF1* (2, 22)*, SF3B1* (2, 22)*, MED12* (2, 22).
Chromatin modification	*KMT2D* (7, 22)*, KDM6A* (7,22)*, ARID1A* (6,22)*, KMT2C* (4, 22)*, ATRX* (3,22)*,TET1* (2, 22)*, EP300* (2, 22)*, NR4A3* (2, 22), *TET3* (2, 22)*, KMT2A* (2,22)*, SETD2* (2, 22).
Cell cycle	*TP53* (11, 22), *RB1 (4*, 22)*, CDKN1A* (3, 22)*, CCND1* (3, 22)*, CDKN2A* (3, 22)*, PAK3* (3, 22)*, BCL2L11* (2, 22)*, USP6* (2, 22).
PI3K-AKT-mTOR	*PIK3CA* (6, 22)*, TEK* (4, 22)*, RHOA* (3, 22)*, IL7R* (2, 22)*, RICTOR* (2, 22)*, FGF19* (2, 22)*, MTOR* (2, 22)*, TSC2* (2, 22)*, AKT2 (2*, 22)*, PTEN* (2, 22).
DNA repair	*BRCA2* (4, 22)*, ATM* (3, 22)*, BAP1* (2, 22)*, ATR* (2, 22).

### Comparison of GAs Between Chinese and Western BUC Patients

**Figure 3:**
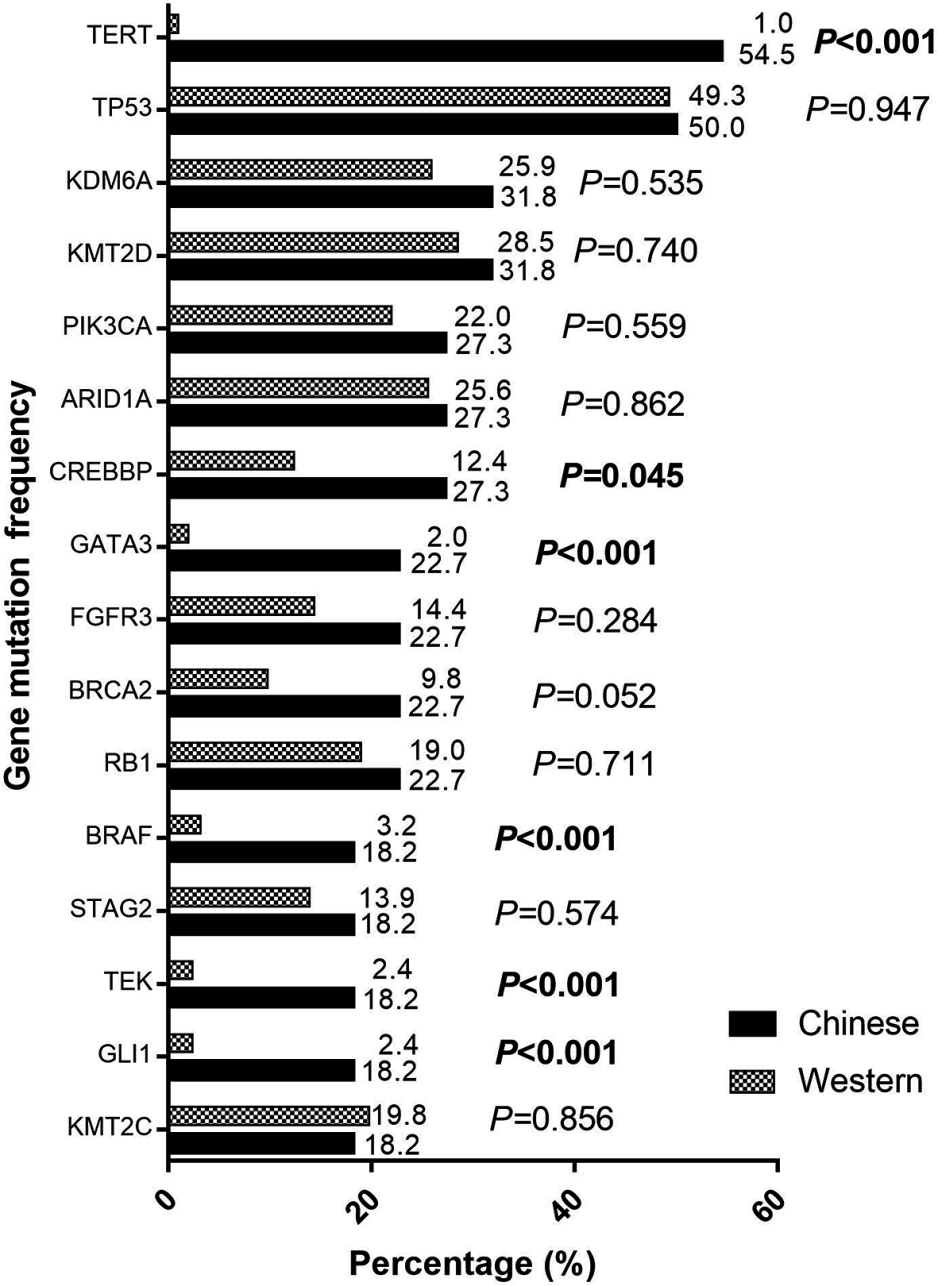
Comparison of the gene mutation frequencies between 22 Chinese and 410 Western patients with bladder urothelial carcinoma. Bolded text: The P-value < 0.05, which was considered as statistically significant.

**Figure 4:**
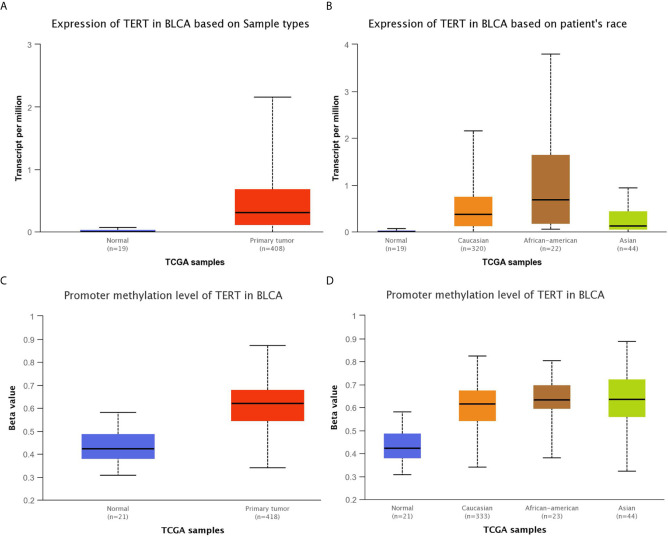
Comparison of the expression and promoter methylation level of TERT between cancer samples and matched normal bladder samples in the TCGA-BLCA cohort. **(A)** The TERT mRNA expression was significantly higher in 408 BLCA samples than in 19 normal bladder samples (P <0.001). **(B)** The TERT mRNA expression was significantly higher in BLCA samples from Caucasian, African–American, Asian than in normal bladder samples (Normal VS Caucasian: P <0.001, Normal VS African–American: P <0.001, Normal VS Asian: P = 0.008, Caucasian VS African–American: P = 0.450, Caucasian VS Asian: P = 0.031, African–American VS Asian: P = 0.024). **(C)** The promoter methylation level of TERT was significantly higher in 418 BLCA samples than in 21 normal bladder samples (P <0.001). The Beta value: 0.5–0.7 was considered hyper-methylation while the Beta value: 0.25–0.3 was considered to indicate hypo-methylation. **(D)** The promoter methylation level of TERT was significantly higher in BLCA samples from Caucasian, African–American, Asian than in normal bladder samples (Normal VS Caucasian: P <0.001, Normal VS African–American: P <0.001, Normal VS Asian: P <0.001, Caucasian VS African–American: P = 0.737, Caucasian VS Asian: P = 0.240, African–American VS Asian: P = 0.604). BLCA, bladder urothelial carcinoma.

The TCGA (BCa) data included the whole genomic sequencing (WGS) results of 410 Western BUC patients. We compared the differences between the top 16 GAs our center and TCGA data by Chi-square test (**Figure 3**). The following gene variation frequencies were significantly higher in the southern Chinese BUC patients: *TERT* (*P <*0.001), *CREBBP* (*P =* 0.045), *GATA3* (*P <*0.001), *BRAF* (*P <*0.001), *TEK* (*P <*0.001) and *GLI1* (*P <*0.001). We subsequently analyzed the expression and promoter methylation level of TERT between cancer samples and matched normal bladder samples in the TCGA-BLCA cohort (**Figure 4**). The TERT mRNA expression (**Figure 4A**) and promoter methylation level (**Figure 4C**) was significantly higher in BUC samples than in normal bladder samples (*P <*0.001). The TERT mRNA expression was significantly lower in Asian BUC patients than in Caucasian (*P* = 0.031) and African–American (*P* = 0.024, **Figure 4B**), whereas the promoter methylation level of TERT in Asian BUC patients was not significantly different from Caucasian (*P* = 0.737) and African–American (*P* = 0.604, **Figure 4D**). We also investigated the genes correlated with TERT in the TCGA-BLCA cohort using the Pearson correlation analysis. The top-five genes were PDCD6 (Pearson correlation coefficient, PCC = 0.64), MRPL36 (PCC = 0.63), NDUFS6 (PCC = 0.60), DUSP23 (PCC = 0.55) and PDZK1 (PCC = 0.53).

### Tumor Mutation Burden Analysis

**Figure 5:**
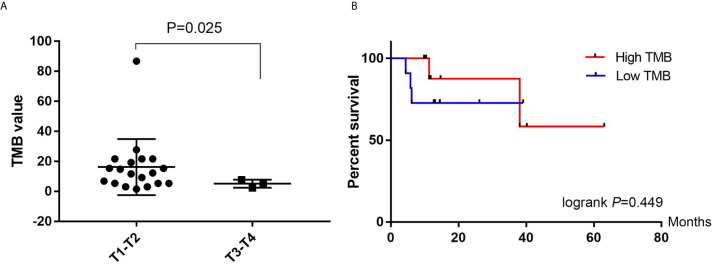
Tumor mutation burden (TMB) analyses. **(A)** Comparison of the TMB value between T1–T2 and T3–T4 patients with bladder urothelial carcinoma. **(B)** Kaplan–Meier plots of recurrence free survival in high TMB (>10.4) and Low TMB (<10.4) patients with bladder urothelial carcinoma. TMB, tumor mutation burden.

The TMB was calculated as the total number of base deletions, substitutions or insertions per mega-base. The TMB value ranged from 1.5 to 86.7 (median, 10.4) among the 22 BUC patients. We compared the TMB value between T1–T2 stages and T3–T4 stages afterwards. The TMB was significantly higher in the T1–T2 group (*P =* 0.025, unpaired *t*-test with Welch’s correction, **Figure 5A**). However, no significant difference of TMB value was found in NMIBC/MIBC (*P* = 0.375) or in different N stages (*P* = 0.543). We obtained the follow-up data from all the 22 involved patients. During the process of follow-up from 9 to 63 months, five patients suffered BUC recurrence and only one died. Hence, we tried to investigate the relationship of TMB value and the recurrence free survival (RFS) of BUC. The Kaplan–Meier plot revealed that BUC patients with low TMB value (<10.4) seemed to have poor RFS time in the first 15 months, however, the statistical difference was not significant (logrank *P* = 0.449, Gehan–Breslow–Wilcoxon test *P* = 0.228, **Figure 5B**).

## Discussion

Deep next-generation sequencing by using large panel is a great progress in the understanding of molecular alterations in cancer. Targeted therapy by using agents on the mutated gene can prolong the survival of patients who own specific drive gene alterations. For example, it was found that patients with metastatic cancer who received targeted therapy had a mean progress free survival (PFS) of 22.9 weeks, compared with 12 weeks in the control group who received standard therapy (17). It is well acknowledged that *HER2* is a crucial target in breast/gastric cancer and the HER2-targeted therapy has been recommended in the first-line clinical guide. However, the BCa heterogeneity poses a severe challenge to adjust drug choices because BCa usually has multi-gene mutation. The WGS results in the TCGA project have facilitated to elucidate the underlying GAs of BCa. Nevertheless, WGS costs much money and time, so it is unpractical to be promoted in clinical practice. We investigated the gene mutation features in BUC patients by a large panel targeted sequencing (450 genes). By comparison with the GAs in Western BCa patients, we aimed to provide new insights into the treatment optimization in southern Chinese patients.

In this study, we found a mass of genes with substitution, truncation and deletion/amplification. The most frequent mutated gene was *TERT*, which was detected in 54.4% patients. Interestingly, the mutation frequency of *TERT* was only 2% in 410 Western BCa patients in the TCGA project. This obvious distinction may be beneficial for small molecule drug development to suit Chinese BUC patients. The *TERT* promoter mutation was found to be correlated with higher level of *TERT* mRNA, and elevated *TERT* mRNA was significantly associated with reduced disease specific survival in two independent BCa patient cohorts (18). We came up with a possible hypothesis: *TERT* mutation will cause the increase of telomerase activity, and then lead to the aggressiveness of BUC cells (19). Researchers used a panel of 23 urothelial cancer cell lines to investigate the *TERT* expression, mutation in urothelial cancer (18). It was reported that TERT mRNA levels were significantly elevated in the BUC cell lines harboring −124 or −146 promoter mutations, while both of the expression level of TERT protein and telomerase activity were higher in those BUC cell lines harboring TERT mutations (18). Other than *TERT*, our results suggested that the gene mutation frequencies of *CREBBP*, *GATA3*, *BRAF*, *TEK* and *GLI1* were also significantly higher in Chinese than Western BUC patients. As for common mutation genes like *TP53*, *KDM6A*, *KMT2D, ARID1A*, our results were in accordance with Western BUC patients. Previous studies reported that mutations in *FGFR3, KDM6A* were more common in NMIBC while mutations in *TP53*, *KMT2D* were more common in MIBC (20–22). Therefore, molecular alterations may help to identify risk subsets and make treatment choices for BUC patients. The two signaling pathways, cell cycle and PI3K-AKT-mTOR were testified by a great deal of studies as key pathways in BUC tumorgenesis, proliferation and invasion (23). GAs in chromatin remodeling and transcription regulation were especially frequent. In this study, we found that the transcription factor and chromatin modification were also two important pathways in the southern Chinese BUC patients.

We also detected gene amplification of *FRS2*, *ERBB2*, *FAS*, etc. in Chinese BUC patients. A recent study reported that *FRS2* duplication mutation was significantly associated with poor prognosis in BUC patients and would act as angiogenesis related drivers (24). Researchers found that *ERBB2* or *FGFR3* mutations were present in 57% of a 105 NMIBC patients cohort (25). Gene fusion alteration is not common in BUC. We detected *RUNDC1/BRCA1*, *MAP4K5/DGKZ*, *RUNDC1/BRCA1* and *SPEN/ARHGEF19* fusion/rearrangement for the first time in BUC. Further PCR and immunohistochemistry experiments should be warranted to verify these possible gene fusion alterations. The TMB value in T1–T2 stage was significantly higher than T3–T4 stage BUC according to our results. Although researchers did not found significant difference of TMB in T1/T2 versus T4/T4 stages in 412 patients, they identified that high low TMB value was significantly correlated with poorer OS (*P* = 0.006) and poorer RFS (*P* = 0.029) in BUC patients (26). GAs may help urologists to select treatment. For example, mitomycin-C, doxorubicin and gemcitabine, were reported more sensitive to BUC with the *TP53* mutation (27). Other researchers verified that BUC patients with high mutation load and activated cell cycle were significantly responsive to an anti-PD-L1 agent (28). It was reported that TERT promoter mutations and chromatin-modifying GAs occurred early in NMIBC tumorigenesis, while ERBB2 or FGFR3 alterations were present in 57% of high-grade NMIBC tumors (29). And somatic ERCC2 mutation was found to be important in improving response to cisplatin-based chemotherapy in MIBC (30). The GAs contributing to NMIBC to MIBC development still need further investigation. And we need more clinical trials to study the actionable mutations and targeted drugs in BUC treatment.

There are some limitations that should be addressed in this study. First, the sample size was small for multivariate analysis, which was unfortunate because there would be potentially some interesting clinical correlations to further analyze. Second, the follow-up time of BUC patients was not long enough, so we did not analyze the overall survival. Third, we will perform further molecular studies based on more BUC tissues, different BUC cell lines, and *in vivo* nude mice models to investigate the potential pathways about the GAs in our future studies. For example, telomere length between cancer and adjacent normal tissue, hTERT assay/telomerase activity analysis, C-circles. Moreover, we are planning to analyze the promoter methylation level and downstream pathways of TERT in BUC patients with and without the TERT variant. Despite the deficiencies above, this study may provide new possibilities on developing new targets for bladder cancer therapy, especially on those unique characteristics that southern Chinese BUC patients possess.

## Data Availability Statement

Publicly available datasets were analyzed in this study. These can be found in the cbioportal website (www.cbioportal.org).

## Ethics Statement

This study was approved by the Ethical Committee of the Third Affiliated Hospital of Sun Yat-Sen University (No. 2019-02-253-01) and written informed consent was obtained from each patient enrolled.

## Author Contributions

All authors made substantial contributions to conception and design, acquisition of data, or analysis and interpretation of data; took part in drafting the article or revising it critically for important intellectual content. All authors contributed to the article and approved the submitted version.

## Funding

This work was supported by grants from the National Natural Science Foundation of China (Nos. 81670688 and 81800666), Guangdong Province Natural Science Foundation of China (Nos. 2016A030313192, 2017A030310414, 2018A030313459 and 2018A0303130330), Guangdong Province Medical Science and Technology Research Foundation Project of China (Nos. A2017365 and 201610010016), the first batch of special grants from the Chinese Post-doctoral Science Foundation (No. 2019TQ0382), foundation from the 3rd Affiliated Hospital of Sun Yat-sen University (YL) and foundation from the Sun Yat-sen University (No. 19ykpy33).

## Conflict of Interest

The authors declare that the research was conducted in the absence of any commercial or financial relationships that could be construed as a potential conflict of interest.
